# Familial aspects of fear of cancer recurrence: current insights and knowledge gaps

**DOI:** 10.3389/fpsyg.2023.1279098

**Published:** 2023-11-15

**Authors:** Aida Faraji, Mohsen Dehghani, Ali Khatibi

**Affiliations:** ^1^Department of Psychology, Shahid Beheshti University, Tehran, Iran; ^2^Centre of Precision Rehabilitation for Spinal Pain (CPR Spine), School of Sport, Exercise and Rehabilitation Sciences, College of Life and Environmental Sciences, University of Birmingham, Birmingham, United Kingdom; ^3^Institute for Mental Health (IMH), School of Psychology, University of Birmingham, Birmingham, United Kingdom; ^4^The Centre for Human Brain Health (CHBH), School of Psychology, University of Birmingham, Birmingham, United Kingdom

**Keywords:** fear of cancer recurrence, family caregiver, close relationships, communication, cancer survivors, cancer-related concerns

## Abstract

Fear of cancer recurrence is fear or worry about cancer recurrence or progress. Fear of recurrence can impact patients’ quality of life and wellbeing. Cancer survivors’ families support them practically and emotionally, making them a vital supplement for official healthcare. Given the well-established important role of the family in dealing with cancer, we compiled the studies that examined the relationship between family-related factors and fear of cancer recurrence (FCR) among cancer survivors (CSs). One of the foremost studies in this field is the FCR model presented by Mellon and colleagues, which included concurrent family stressors and family-caregiver FCR as factors linked to survivor FCR. Our goal was to prepare the ground for a family-based model of FCR that is more comprehensive than the one proposed by Mellon et al. sixteen years ago. The studies included those with samples of adult cancer survivors from different regions of the world. Most of the studies we reviewed are cross-sectional studies. We categorized family-related factors associated with survivor FCR into partner-related factors, including subgroups of disclosure to partner, cognitions of partner, and partner’s sources of support; parenthood-related factors, including having children and parenting stress; family-related factors, including living situation, family history of cancer, family’s perception of the illness, and family characteristics; and social interactions including social support, disclosure, social constraints, and attitudes of others. This review sheds light on how significant others of cancer survivors can affect and be affected by cancer-related concerns of survivors and emphasizes the necessity of further investigation of family-related factors associated with FCR.

## 1 Introduction

Cancer has become one of the most prevalent conditions that impact people worldwide, but the number of survivors is increasing with improvements in treatments and care (Cancer Today, 2020). For instance, nearly 20 million new cancer cases were diagnosed worldwide in 2020 (Sung et al., 2021). However, for some types of cancer, a significant percentage of patients are expected to survive. For example, global 5-year survival rates for breast and prostate cancer, two of the most common cancers, exceed 90% (Nardin et al., 2020; Subudhi, 2023). While it is great news that many people are now surviving a once-deadly disease, research shows that Cancer survivors (CS) experience a wide range of problems caused by cancer and its treatments, including physical, psychosocial, spiritual, and existential issues, some of which persist for years (Institute of Medicine and National Research Council, 2006). Future uncertainty and fear of cancer recurrence are among the most common difficulties that CSs and their caregivers experience (Institute of Medicine and National Research Council, 2006; Jefford et al., 2008).

Fear of cancer recurrence (FCR) is defined as fear or worry about cancer recurrence or progress (Lebel et al., 2016) and is experienced at moderate to high levels by 59% of CSs (Luigjes-Huizer et al., 2022). FCR can motivate survivors to promote healthy behaviors to adapt to their new situation (Park and Gaffey, 2007). On the other hand, lower quality of life, more psychological distress, increased use of healthcare, and increased healthcare costs are downsides to FCR (Thewes et al., 2012; Simard et al., 2013; Lebel et al., 2014, 2016; Jimenez et al., 2017; Champagne et al., 2018; Hall et al., 2018).

Family support is one of the sources that help the survivors deal with their challenges to such a degree that it has been known as a vital supplement for official healthcare (Nijboer et al., 1998; Haley, 2003; Koltai et al., 2018). The family becomes a part of the caregiving team for patients. They get involved in a wide range of issues, from symptom management to problems related to hospitalization and dealing with financial, autonomy, psychological, and social issues (Effendy et al., 2015). Families of CSs support them emotionally by reassuring and consoling, expressing love and affection, being present, distracting the patient from cancer, and practically by accompanying them to the hospital for examinations, treatments, support with household chores, etc. (Vrontaras, 2018). The impact of the family on patients’ adaptation to their new situation and how they cope with the condition goes beyond simply being a support network. For example, spouse-caregivers with higher emotional distress early after diagnosis significantly decrease patient adaptation to cancer a year later (Park et al., 2010). Many studies tried to model the family’s contribution to cancer survivorship, hoping that the model can help design interventions to improve patient’s quality of life and adaptation.

Exploring familial aspects of fear of cancer recurrence needs to be a priority in psycho-oncology because this form of health anxiety is increasing due to the increasing number of survivors. Moreover, research has shown that multiple dimensions of both caregiver and patient well-being, including role adjustment, mental health, quality of life, and psychological distress are interrelated (Northouse et al., 2000; Chen et al., 2004; Bambauer et al., 2006; Kim and Given, 2008). Following the lead of these studies, fear of cancer recurrence should be seen as a factor influenced by caregivers, which usually means family members. As a result of our deepened understanding of how family members influence survivor FCR, we can educate families on how to alleviate FCR in survivors. Further, we would be able to design psychological interventions that could involve family members in the therapy for those with elevated FCR. It should arguably be so because family members are as involved as the survivors with the emotional impacts of cancer.

Mellon et al. (2007) suggested a family-based model of FCR, which was influenced by the resilience model (McCubbin and McCubbin (1996). This intricate family resilience model describes the link between stressors and increased demands for family adaptation. In short, according to this model, stressor events and the pile-up of demands affect family meaning and schema, situational appraisal, family resources, and social support, either through family type or directly, which in turn influence family problem-solving and coping skills. Finally, family problem-solving and coping skills are directly linked to family adaptation (McCubbin and McCubbin, 1996). Inspired by this model, Mellon et al. (2007) proposed their model of FCR in which several individual factors (including age, education, sex, and race), stressors (including concurrent family stressors and illness-related stressors), and family resources (including family hardiness and social support) affected fear of recurrence in survivors and family members through their illness representation. In this model, there is a bidirectional relationship between CS and family members’ fears. Testing their model on a sample of CSs and their family members, they presented a revised model (Mellon et al., 2007): individual factors, stressors, and illness representation affect the CS and family members’ FCR directly, and the relationship between the CS and family member’s fear is bidirectional, as it was in the initial model. Mellon et al. (2007) have acknowledged that many other factors can be added to their model. Since Mellon et al. (2007) model was proposed, several studies have looked into family-related factors, such as the type of relationship and social context, associated with survivors’ FCR that may suggest alterations to the original model.

This narrative review aims to examine the studies investigating the link between fear of recurrence in patients and family members and how their relationship and social context impacted patients’ fear of recurrence. We have categorized family-related factors into partner-related, parenthood-related, family-related, and social interactions.

## 2 Study selection

The search was conducted on PubMed and Google Scholar. Relevant keywords in the search included fear of cancer recurrence, FCR, fear of cancer progression AND family, caregiver, spouse, partner, parent, mother, father, child. During reviewing papers, if a new keyword was discovered (e.g., disclosure), it was searched to include potentially relevant articles. References and citations have been explored for relevant publications. Among the articles, those that assessed FCR in adult survivors and contained variables or themes that involved the social circle of cancer survivors were selected for the review. The search was conducted in April 2023 and included peer-reviewed articles published in English between 2001 and 2023. A total number of 38 publications met these criteria and were included in this review. Table 1 presents an overview of these studies.

**Table 1:** Studies included in the current review examining family-related factors related to FCR and summary of their findings.

Publication	Study design	Measures/questionnaires	Cancer type	Findings/associations
Acheampong et al. (2020)	Cross-sectional	FCR-4	Breast	Having children t(84.43) = 4.35, *p* < 0.001
Aghdam et al. (2014)	Descriptive-correlational	FoP-Q-SF	Leukemia Gastro-Intestinal Breast Lung	“Fear of children contracting cancer” as the highest-rated item in the FoP-Q-SF “Worry about family” as the second highest-rated item in the FoP-Q-SF
Arès et al. (2014)	Cross-sectional	CARS^1^	Breast	Having children (*F*(1, 738) = 9.60, *p* = 0.002, partial *η*2 = 0.013). Parenting stress (*β* = 0.18, t (515) = 3.25, *p* = 0.001)
Bergman et al. (2009)	Prospective observational cohort	MAX-PC^2^	Prostate	Having a partner (PE = 5.79, *p* = 0.03)
Boehmer et al. (2016)	Cross-sectional	The self-report measure developed by Northouse (1981)	Breast	Caregiver FCR (*t* = 3.15, *p* = 0.0017) Survivor-caregiver co-residence (*t* = 3.44, *p* = 0.0006) Caregiver social support (*t* = −2.57, *p* = 0.0102) Caregiver seeking counseling (*t* = 4.98, *p* = 0.0001)
Chien et al. (2018)	Prospective repeated-measures & experimental with random assignment	MAX-PC	Prostate	Spouse’s religious beliefs (*β* = −0.211, *p* = 0.027) Living with the extended family (*β* = −0.232, *p* = 0.033)
Cohee et al. (2017)	Cross-sectional	CARS	Breast	Cognitive processing as a mediator between social constraints (*a* = 0.631, *p* = 0.001) and FCR (*b* = 0.292, *p* = 0.001)
Custers et al. (2017)	Cross-sectional	CWS^3^	Breast	Having children (t(445) = −2.37,*p* = 0.018)
Dumalaon-Canaria et al. (2018)	Cross-sectional	CARS	Breast	Family history of cancer (*r* = 0.143, *p* = 0.011)
Dunn et al. (2015)	Longitudinal	Four items from the QOL-PV^4^	Breast	Living alone (SE = −0.654, *p* = 0.270) Distress of illness to family (SE = 0.154, *p* = 0.052)
Galica et al. (2020)	Qualitative	Semi-structured review	Ovarian	Family support as the best resource for dealing with FCR
Götze et al. (2019)	Cross-sectional cohort	FoP-Q-SF^5^	Breast Gynecological Kidney Hematological Colon Skin Head and Neck Prostate	“Worry about family” as one of the top fears underlying FCR
Halbach et al. (2016)	Prospective, multicentre cohort-study	FoP-Q-SF	Breast	Having children (*r* = 3.26, *p* = 0.017)
Hamama-Raz et al. (2022)	Qualitative	semi-structured in-depth interviews	Cervical	Three central themes of FCR: No longer resilient,” “To be afraid in a dyad,” “And what if the disease comes back and I die?”
Hanprasertpong et al. (2017)	Prospective cross-sectional	FoP-Q-SF	Cervical	“Worry about family” as one of the top fears underlying FCR
Hu et al. (2021)	Cross-sectional	FoP-Q-sf	Multiple Myeloma	Partner FCR (*r* = 0.614, *p* < 0.01) Family hardiness (*r* = −0.267, *p* < 0.01) Social support (*r* = −0.287, *p* < 0.01)
Humphris et al. (2019)	Mixed-methods observational	FCR-7	Breast	Emotional talk with therapeutic radiographer (*β* = −0.514, *p* = 0.011)
Iglesias-Puzas et al. (2022)	Cross-sectional	FCR-7	Non-Metastatic Melanoma	Having a family history of cancer (1.9 times higher)
Johnson Vickberg (2001)	Qualitative	Semi-structured interview	Breast	Social support
Koch-Gallenkamp et al. (2016)	Cross-sectional	FoP-Q-SF	Breast Colorectal Prostate	Social support (odds ratio = 2.13, 95% confidence interval = 1.78–2.55)
Lai et al. (2019)	Qualitative	purposive sampling technique	Breast	FCR themes: “Trapped in insecurity,” “Suffering in silence,” and “Pretending as if nothing has happened.”
Lebel et al. (2013)	Cross-sectional	CARS	Breast	Having children (*F* = 6.64, *p* < 0.001)
Liu et al. (2022)	Cross-sectional	FoP-Q-SF	Lung	Social support (*r* = −0.255, *p* < 0.01)
Mehnert et al. (2009)	Cross-sectional	FoP-Q-SF	Breast	Motherhood (*d* = 0.14, *p* = 0.05)
Mehnert et al. (2013)	Prospective multicentre cohort	FoP-Q-SF	Gynecological Head and Neck Skin Colon/Rectum Lung Hematological Neoplasia	Social support (*r* = −0.16, *p* < 0.001) Detrimental interactions (*r* = 0.37, *p* < 0.001)
Melchior et al. (2013)	Cross-sectional	FoP-Q-SF	Breast	Having children (*b* = 0.159, *p* = 0.089)
Mellon et al. (2008)	Cross-sectional	CWS	Breast and/ or Ovarian	Having a partner (*b* = 0.11, *p* = 0.03) Family history of cancer (*b* = 0.03, *p* = 0.9)
Muldbuecker et al. (2021)	Cross-sectional	FoP-Q-SF	Prostate Laryngeal Breast	Partner FCR Prostate Cancer (*r* = 0.51, *p* = 0.001) and Breast Cancer (*r* = 0.31, *p* = 0.001)
Perndorfer et al. (2019)	Longitudinal	FCRI^6^	Breast	Protective buffering (*r* = 0.24, *p* = 0.001)
Perndorfer et al. (2022)	Longitudinal	FCRI	Breast	Partners’ sleep quality (*b* = −0.85, *p* = 0.001) Partners’ sleep onset latency (*b* = 0.23, *p* = 0.022)
Rincones et al. (2021)	Systematic review of literature	Various	Testicular	Having a partner (NA)
Sawma and Choueiri (2022)	Cross-sectional	FCRI–SF^7^	Breast	Balanced flexibility (*b* = −0.67, *p* < 0.001) Quality of communication (*b* = −0.33, *p* = 0.004) Chaotic family functioning (*b* = −0.49, *p* = 0.001)
Şengün İnan and Üstün (2019)	Qualitative	Semi-structured interviews	Breast	Motherhood Social support Stigma and the negative attitudes of others, especially spouses
Shen et al. (2022)	Cross-sectional	FCRI-SF	Acute Leukemia	Social constraints (*r* = 0.362, *p* < 0.01)
Shi et al. (2022)	Quasi-experimental	FoP-Q-SF	Cervical	The quality of communication within the family (*t* = 6.169, *p* < 0.001)
Shin et al. (2022)	Cross-sectional	FCRI	Stomach	Social support (*b* = −0.190, *p* < 0.001)
Singh-Carlson et al. (2013)	Qualitative	Semi-structured interviews	Breast	Younger women experiencing FCR relating to uncertainty around their future, middle-aged women related to what would happen to their children and older women not being troubled by FCR
Soriano et al. (2018)	Longitudinal	FCRI	Breast	Spouse responsiveness (week2: coefficient estimate = 0.456, *p* < 0.01; week3: coefficient estimate = −0.421, *p* < 0.01)
Soriano et al. (2019)	Cross-sectional & longitudinal	FCRI	Breast	Spouse threat sensitivity (estimate coefficient = −0.109, *p* < 0.001)
Soriano et al. (2021)	Longitudinal	FCRI & CARS	Breast	Social constraints (estimate coefficient = 1.117, *p* = 0.05) Protective buffering (estimate coefficient = 1.102, *p* < 0.001)
Steele et al. (2007)	Cross-sectional	Specific questionnaire designed for this study	Colorectal	Having children younger than 21 (NA)
Thewes et al. (2016)	Qualitative	semi-structured interview	Breast	Social support Talking to unsupportive or negative people about FCR Disclosure to friends and family and support groups “Worry about family” as one of the top fears underlying FCR
Uner and Korukcu (2021)	Qualitative	semi-structured interview	Cervical	“Worry about family” as one of the top fears underlying FCR
van de Wal et al. (2017)	Cross-sectional	CWS	Prostate	Partner FCR (*r* = 0.44, *p* < 0.001)
Wijayanti et al. (2018)	Cross-sectional	FCRI	Gynecological	Family history of cancer (*t* = 5.53, *p* = 0.001) Social support (self-esteem support (*F* = 32.33, *p* < 0.05), appraisal support (*F* = 34.14, *p* < 0.05), and belonging support (*F* = 28.28, *p* < 0.01))
Wu et al. (2019)	Longitudinal	Single-item	Prostate	Spouse FCR (Baseline (ICC^8^=0.34, *p* = 0.004) and 6 months (ICC = 0.26, *p* = 0.02))
Xu et al. (2019)	Longitudinal	Single-item	Breast	Disclosure of positive information via patient’s perception of positive information (*B* = 0.130, *p* < 0.01; B = -0.315, *p* < 0.001)
Yeung and Lu (2022)	Cross-sectional	Single-item	Breast	Social constraints (*r* = 0.31, *p* < 0.001)
Zheng et al. (2022)	Cross-sectional	FoP-Q-SF	Lung	Social support (*r* = −0.416, *p* < 0.000)
Zhong et al. (2022)	Cross- sectional	FoP-Q-SF	Glioma	Perceived social support (*r* = −0.504, *p* < 0.05)

^1^Concerns About Recurrence Scale.

^2^Memorial Anxiety Scale for Prostate Cancer.

^3^Cancer Worry Scale.

^4^Quality of Life—Patient Version.

^5^Fear of Progression Questionnaire--short form.

^6^Fear of Cancer Recurrence Inventory.

^7^Fear of Cancer Recurrence Inventory—Short Form.

^8^Intraclass Correlations.

## 3 Synthesis of findings

### 3.1 Partner-related factors associated with fear of cancer recurrence

To begin with, having a partner or not is one of the factors that may impact the level of FCR in CSs. Partnered men diagnosed with prostate or testicular cancer have shown significantly less FCR than single ones (Bergman et al., 2009; Rincones et al., 2021). In contrast, married women had more cancer worries than non-married women in a sample of women with breast or ovarian cancer (Mellon et al., 2008). These studies may not be comparable due to differences in design, scales used, different definitions of relationship and the different nature of prostate, testicular, breast, and ovarian cancers. Also, the mentioned studies are exceptions in the relationship between FCR and marital status since most studies have found no significant relationship between the two variables (Northouse, 1981; Leake et al., 2001; Llewellyn et al., 2008; Simard et al., 2010; Custers et al., 2017; Dumalaon-Canaria et al., 2018; Lebel et al., 2018; Starreveld et al., 2018; Thewes et al., 2018). However, a plausible explanation for the contrasting results could be that women are traditional caregivers in most cultures, and this makes them a relieving caregiver when their partner is ill. In these circumstances, they become care receivers, and the same fact makes them feel like a burden on their partners. A study finds gender role conditioning to be an underlying factor for women assuming the role of caregiver for themselves (Guberman et al., 1992). In addition to gender differences, it would be beneficial to analyse the data controlling for age since it is possible that age would be a moderator in the relationship between marital status and FCR.

In a qualitative study investigating the meaning of FCR for cervical cancer survivors, one of the main themes that emerged was “to be afraid in a dyad,” which refers to FCR being the sort of challenge that is discussed with a partner and dealt with by getting help from partner’s resources (Hamama-Raz et al., 2022). In this study, communication with partners seems to be the main distinction between women who feel alone in their survivorship experience and those who do not (Hamama-Raz et al., 2022). Several quantitative studies confirm the significant relationship between open communication with a partner and survivor FCR. Protective buffering, which is defined as “efforts to protect one’s partner from upset and burden by concealing worries, hiding concerns, and yielding to the partner to avoid disagreements” (Manne et al., 2007), is a construct measured by the extent to which CSs or their partners engage in specific behaviors to deal with cancer-related issues. Likewise, the social constraints are constructs reflecting the perception that one cannot share cancer-related thoughts, concerns, or worries with one’s spouse on account of his/her disinterest, unavailability, or disapproval (Lepore and Revenson, 2007). Higher protective buffering and social constraints of CSs have been shown to predict their increased FCR in longitudinal studies, although one’s protective buffering or social constraints do not affect his/her partner’s FCR (Perndorfer et al., 2019; Soriano et al., 2021). Cognitive processing is suggested to mediate the relationship between social constraints and FCR, according to Cohee et al. (2017).

Capitalisation, which describes the process of disclosing positive events to a close other (attempt), whose response is perceived as genuine and enthusiastic (perceived partner responsiveness, hereafter termed responsiveness) (Langston, 1994; Gable and Reis, 2010), is another way of looking into couple communication. A study by Soriano et al. (2018) has examined the relationship between capitalisation and FCR around the first mammogram post-diagnosis, from which inconsistent results have emerged: although they had hypothesized that both attempts and responsiveness would buffer FCR, attempts never significantly predicted lower FCR and responsiveness only predicted lower FCR after the mammogram. Disclosure of positive information by spouse is also another factor influencing FCR according to a longitudinal study: spouses’ disclosure of information that was communicated in a positive manner (i.e., supportive, inclusive, and concerned manner) has shown to be linked to breast cancer survivors’ decreased FCR via breast cancer survivors’ perceptions of positive information (Xu et al., 2019). The results of the studies that emphasize the effect of within-couple communication on FCR can be justified by the social-cognitive processing model, which suggests that sharing concerns with a close other is an adaptive response to adversity since it facilitates cognitive processing (Lepore, 2001; Lepore and Revenson, 2007). So, any variable that refers to openness to communication may be related to FCR, while withholding worries hinders cognitive processing, thus impends adjustment and maintains FCR (Lepore, 2001; Lepore and Revenson, 2007). Within-couple communication relationship with survivor FCR has been supported by several studies, most of which are longitudinal ones, making the data more reliable and the suggested relationship more likely to be a cause-and-effect relationship. However, all quantitative studies that suggest this link have been conducted on women diagnosed with breast cancer, which hinders the generalizability of the results. Women diagnosed with breast cancer are more concerned with some cancer-related problems than women diagnosed with other cancer types, which probably makes the nature of their fears about recurrence different from other CSs. For example, women with breast cancer experience higher sexual dysfunction [including abnormalities in sexual desire, arousal, lubrication, satisfaction, orgasm, and dyspareunia (Boquiren et al., 2016)] in comparison with women with other cancer types (Jing et al., 2019), which probably causes more worries concerning sexuality. Worries about the sexual consequences of cancer and its treatment is a topic to discuss with a partner, so couples’ communication may be quite beneficial for women with breast cancer but not as helpful for women with other cancer types. Future research is needed to investigate the content of discussions about FCR with partners (e.g., surrounding which items of CARS) and compare the effect of discussing each specific concern on FCR. Furthermore, no study has been conducted on men regarding the relationship between couples’ communication and FCR.

Some cognitions of CSs’ partners are also associated with survivor FCR. In a qualitative study on Turkish breast cancer survivors, an identified trigger of FCR was stigma and negative attitude of spouses (Şengün İnan and Üstün, 2019). Threat sensitivity, which reflects individual differences in the general tendency to attend to, behaviourally and emotionally respond to, and avoid threatening negative stimuli (Carver and White, 1994), is also characteristic in partners that can influence survivor FCR. When FCR peaks in the first mammogram post-diagnosis in breast cancer survivors and their spouses, spouse threat sensitivity, not CS’s, predicts longer recovery from FCR peak in both CS and spouse (Soriano et al., 2019). However, spouse threat sensitivity could not predict reactivity or patient FCR on mammogram day (Soriano et al., 2019). Like attitudes toward cancer and threat sensitivity, having religious beliefs or not as the partner of a CS is linked to survivor FCR. In a sample of Taiwanese prostate cancer survivors and their partners, CSs whose partners had religious beliefs reported less FCR than those with partners without religious beliefs (Chien et al., 2018). Of course, we are unsure which element or function of religion causes this link. The most studied construct concerning survivor FCR in a relationship context is partner FCR. Several studies have shown a positive association between survivor FCR and partner FCR among different sexes, cancer types, countries, and FCR scales (Boehmer et al., 2016; van de Wal et al., 2017; Hu et al., 2021; Muldbuecker et al., 2021). Not much is known about the causality that may lay under this association. However, in the study by Boehmer et al. (2016), partners’ FCR directly affected survivors’ FCR, while survivors’ FCR did not affect partners’ FCR. Likewise, spouse FCR 6 months after treatment showed a significant association with patient FCR a year after treatment, but no trends toward patient FCR being correlated with later spouse FCR emerged, which indicates that it may be spouse FCR, that influences patient FCR, not vice versa (Wu et al., 2019). The effect of partner cognitions on survivor FCR seems only natural since FCR is coped with in a dyad (Hamama-Raz et al., 2022), and partners are frequently cited as the most important confidants (Figueiredo et al., 2004); when a CS counts on someone as her companion in adversity, the companion’s thoughts on the matter gains importance and affects the way the CS thinks and feels about her experience.

Another category of partner-related factors associated with survivor FCR is the partner’s source of support. CSs whose partners have higher social support seem to have lower FCR, while those who seek counseling have higher FCR (Boehmer et al., 2016). A possible explanation could be that seeing a partner receiving support from more conventional sources (e.g., family and friends) is a sign of them handling the situation well, whereas seeking help from a professional signifies a crisis or an overwhelming situation. According to this potential explanation, CSs are less fearful about cancer relapse or progression when their partner is handling the situation well.

Not many studies have worked on factors associated with FCR in the relationship context that are potential consequences of survivor FCR. But, survivor FCR is associated with a partner’s reduced sleep quality and greater sleep onset latency in a sample of American couples coping with early-stage breast cancer (Perndorfer et al., 2022). Speculation about why this association exists can be that partners are emotionally and physiologically sensitive to CSs’ worries to a high degree.

### 3.2 Parenthood-related factors associated with fear of cancer recurrence

Not much research has been done on how being a parent affects FCR. But several studies suggest that motherhood is a trigger of FCR, a factor affecting the nature of FCR, and associated with higher FCR (Mellon et al., 2008; Mehnert et al., 2009; Lebel et al., 2013; Melchior et al., 2013; Arès et al., 2014; Halbach et al., 2016; Custers et al., 2017; Şengün İnan and Üstün, 2019; Acheampong et al., 2020). Further, Steele et al. (2007) found that women younger than 21 with children experience higher levels of FCR. Similarly, Singh-Carlson et al. (2013) found that younger women commonly experienced FCR relating to uncertainty around their future, whereas, for middle-aged women, the FCR centred around what would happen to their children and older women were not troubled by FCR. A more specific construct concerning parenting and how it might interact with FCR is investigated by Arès et al. (2014), who found that parenting stress increases FCR. In the study by Arès et al. (2014), young breast cancer survivors who had children Arès et al. (2014) also reported that breast cancer interfered more with their intimate lives than childless CSs. These findings pieced together imply that the heightening effect of motherhood on FCR comes from women’s responsibility for their children. Cancer recurrence means having to go through intense treatment, having less time to spend with their children; an increase in the possibility of their death, leaving their children motherless; and an increase in the responsibilities expected from their adolescent due to his/her mother being hospitalized or debilitated. Consequently, women worry about cancer recurrence more if they have children they should care for.

A study by Aghdam et al. (2014) on Iranian CSs (male and female) showed that fear of children contracting cancer is the highest-rated item in the Fear of Progression Questionnaire. To our knowledge, no study has specifically examined the relationship between fatherhood and FCR.

### 3.3 Family-related factors associated with fear of cancer recurrence

Some quantitative and qualitative studies on samples from various cultures, cancer types, and ages have identified “worry about family” as one of the top fears underlying FCR (Aghdam et al., 2014; Thewes et al., 2016; Hanprasertpong et al., 2017; Götze et al., 2019; Uner and Korukcu, 2021). Further, Uner and Korukcu (2021) have found the basis for fear of death to be fear of leaving their loved ones alone among young Turkish CSs suspected of new cancer. Considering this finding, it could be estimated that some CSs’ worry for their families is manifested in items or themes other than “fear of family being affected by cancer,” and family has even greater importance in their mind than the results that research shows.

The living situation of CSs seems to influence their FCR levels. Survivors who do not live alone experience higher FCR (Dunn et al., 2015) and survivor-caregiver co-residence seem to increase survivors’ FCR (Boehmer et al., 2016). Although these results have come solely from female breast cancer survivors from the USA, a population probably not representative of all CSs, and they used unconventional measurements for FCR, they give us a cue for further investigation of how CSs perceive the influence cancer has on their relationships with people around them. Another study concerning the living situation of cancer survivors and FCR was carried out on an entirely different population. Chien et al. (2018) found Taiwanese prostate cancer survivors with their partners, children, and grandchildren to have lower FCR than those with only their partners. The contrasting results of these studies emphasize sex and cultural differences. For example, it may be the case that CSs in American culture, an individualistic society that emphasizes autonomy, feel like a burden on the people they live with, while CSs in Taiwanese culture, a collectivist society that counts taking care of elderly family members as a duty, feel quite comfortable with getting as much help as they need from their extended families. Alternatively, the reason for the difference between the studies of Boehmer et al. (2016) and Dunn et al. (2015) and the study of Chien et al. (2018) may be partially again due to women being traditional caregivers, who feel like they should not be care receivers.

Another family-related factor associated with FCR is a family history of cancer. Having a family history of cancer seems to increase FCR, as seen in American women with breast/ovarian cancer, Australian women with breast cancer, Spanish melanoma patients and Indonesian women with gynaecological cancer (Mellon et al., 2008; Dumalaon-Canaria et al., 2018; Wijayanti et al., 2018; Iglesias-Puzas et al., 2022). More research is needed to make clear how a family history of cancer may affect a CS’s perception of cancer, recurrence, and caregiving. However, a plausible explanation is that maybe CSs with a family history of cancer are more cognizant of the difficulties accompanied by cancer, or they think that their family resources are drained after dealing with cancer once.

Similar to the study by Mellon et al. (2007), which relates the meaning of illness to family members to FCR, Dunn et al. (2015) showed that distress of illness in the family is associated with FCR. This data means there may be a cause-and-effect relationship or a bidirectional interaction between how family members perceive and feel about cancer and survivor FCR. This is very likely since there is evidence of CSs mentioning the attitude of people around them, especially their partners, as affecting their FCR (Thewes et al., 2016; Şengün İnan and Üstün, 2019).

Some family characteristics have been linked to FCR, expected concerning how families communicate and handle difficulties. Family hardiness, balanced flexibility and the quality of communication within the family are associated with decreased FCR (Hu et al., 2021; Sawma and Choueiri, 2022; Shi et al., 2022). On the other hand, chaotic family functioning increases FCR levels, while cohesion, disengagement, enmeshment, and family satisfaction does not seem to impact the severity of FCR (Sawma and Choueiri, 2022). These studies only include Chinese and Lebanese CSs, with the majority of them being women. So, with regard to differences in the role of the family in Eastern cultures and Western ones, it seems necessary to compare the relationship of these constructs with FCR in various cultures. But according to what we know until now, it could be said that FCR is more effectively curbed in families that are more flexible in their roles, more resilient to stress, better in problem-solving, cooperation, and open communication, and feel more in control of difficulties.

Family support is the best resource for dealing with FCR for some CSs (Galica et al., 2020). However, not all people feel comfortable to discuss their worries about cancer with their family members. In a Taiwanese qualitative study on women who have been diagnosed with breast cancer in the last 2 years, three themes emerged for FCR: “Trapped in insecurity,” “Suffering in silence,” and “Pretending as if nothing happened” (Lai et al., 2019). These women did not mention their feelings surrounding cancer to their families to maintain family balance and continued to perform their roles in the family as before the cancer (Lai et al., 2019).

### 3.4 Social interactions’ relationship with FCR

Some researchers prefer not to limit social interactions that relate to FCR to partners and family members. They, thus, explore the social support construct in their studies, which refers to the support that an individual gets from family, friends, and other people she may feel close to, such as colleagues, neighbors, and her medical team. This expansion makes sense since many people feel closest to their significant others who are not family members or partners. In this case, these significant others probably play a bigger role in supporting the CS. Also, this type of research’s findings apply to a family context. Therefore they are included in the current review.

Many studies from different countries and various cancer types have shown that CSs with higher social support experience lower FCR (Mehnert et al., 2013; Koch-Gallenkamp et al., 2016; Şengün İnan and Üstün, 2019; Hu et al., 2021; Liu et al., 2022; Shin et al., 2022; Zheng et al., 2022; Zhong et al., 2022). Likewise, a significant negative relationship has been observed between the number of significant others and FCR, along with a strong link between the number of significant others CSs identify as understanding her health concerns and FCR (Northouse, 1981). Fear of loneliness and fear of relying on strangers for daily activities in case of cancer recurrence has also been mentioned as an important part of FCR, which confirms the idea that social support is a determining factor in FCR (Götze et al., 2019; Şengün İnan and Üstün, 2019). How social support may link to lower FCR can have multiple answers, one of which is extracted from qualitative studies showing that CSs find social support an effective coping strategy in the face of FCR, using it more than any other coping strategy (Johnson Vickberg, 2001; Thewes et al., 2016). Another way of social support influencing FCR can be through resilience, as suggested by Zhong et al. (2022).

An important part of social support, which researchers address, is communication. Failure to disclose is reported to be inversely correlated to social support and positively associated with receiving unsupportive responses (Figueiredo et al., 2004). There seem to be two sides to disclosing FCR-related thoughts and emotions: In a study by Thewes et al. (2016), some participants from all levels of FCR reported that disclosure to friends, family, and support groups provided opportunities for emotional ventilation and mutual support, while some others found it anxiety-producing because of the perceived impact of these discussions on others. Due to the adverse effect that talking about cancer-related worries may have on others, some CSs withhold these worries in order to protect family and friends (Şengün İnan and Üstün, 2019). Taking into account worries about the effect of cancer talk on family and friends, some patients opt to disclose their worries to professionals, which seems to reduce FCR: emotional talk of breast cancer patients during their second review appointment with their therapeutic radiographers is negatively associated with follow-up FCR, which is measured 6–8 weeks after the end of treatment (Humphris et al., 2019). We can again interpret these associations in the social-cognitive processing model framework, which suggests that sharing concerns with a close other is an adaptive response to adversity since it facilitates cognitive processing (Lepore, 2001; Lepore and Revenson, 2007).

In its broad sense, social constraints not limited to spouses discussed previously can increase FCR (Shen et al., 2022; Yeung and Lu, 2022). Mediating factors between social constraints and FCR for which evidence has emerged are illness perception, self-stigma, bodily pain, and ambivalence over emotional expression (Shen et al., 2022; Yeung and Lu, 2022). Both studies that link social constraints and FCR have been done on Chinese samples, which may limit the generalizability of these findings. For instance, the Chinese tend to translate their inhibited emotions into somatic symptoms (e.g., pain), which means that the mediating effect of bodily pain in the link between social constraints and FCR could be limited to the Chinese culture (Mak and Zane, 2004). So, in order to generalize these findings, more research has to be done in other cultures, along with a quest for other possible mediating factors in the relationship between social constraints and FCR, such as cognitive processing, coping behaviors, optimism, self-efficacy, and threat appraisal.

Although getting help from their support circle can assist CSs in reducing their FCR, sometimes, this support circle triggers FCR. Women from a study by Thewes et al. (2016) identified talking to unsupportive or negative people as an ineffective coping strategy for FCR, and women from a study by Şengün İnan and Üstün (2019) think that behaving as if they were still ill after treatment by people around them was a trigger for FCR. Moreover, detrimental interactions (including over-protective behavior, dismissive, conflictual behavior patterns, and pessimism) have been identified to predict higher FCR (Mehnert et al., 2013). Hence, disclosing cancer-related thoughts and feelings to others does not decrease FCR unconditionally, and potential harm underlies some social interactions.

## 4 Discussion

This narrative review paper aimed to categorize family-related factors associated with survivors’ FCR under partner-related factors, including subgroups of disclosure to partner, cognitions of partner, and partner’s sources of support; parenthood-related factors, including having children and parenting stress; family-related factors, including living situation, family history of cancer, family’s perception of the illness, and family characteristics; and social interactions including social support, disclosure, social constraints, and attitudes of others.

The results of this narrative review of quantitative and qualitative literature signify a variety of family-related factors greatly affecting survivors’ fear of recurrence. Although relatively few studies account for familial aspects of FCR, the results promise at least some family-related factors to account for FCR variations. This means that in the near future, we may be able to have a family-based model of FCR and base a family-oriented intervention on it.

We chose narrative review over systematic review because the inclusion and exclusion criteria required by systematic review limit the breadth of the papers included. Some reviewed papers that offer insightful contributions to the field have used unconventional scales, do not have a rigorous methodology, or are unsuitable for quantitative synthesis. We aimed to bring attention to all the family-related constructs correlated with FCR to lay the grounds for original research that models potential contributing factors. So, we refrained from a methodology that would dismiss a paper with relevant findings that could inspire us to find other constructs in the same category. Moreover, not many papers explore the familial aspect of FCR; therefore, excluding a few papers affects the take-home message of this review more than it usually does.

The most prominent feature of this literature review is that it gathers together studies from different countries, various cancer types, and various methodologies that have one thing in common, which is the key to looking at FCR in a new way: accounting for family resources. This review intends to lead researchers to look for resources for improving the mental health of survivors in the family and even community instead of looking for what resources an individual has. No single person can bear the burden of dealing with cancer and its consequences alone since this disease makes them physically and mentally vulnerable.

The obvious limitation of this study is that it has yet to use rigorous methodologies. Thus, regardless of the authors’ attempts to stay impartial, it is inclined to bias.

**Figure 1:**
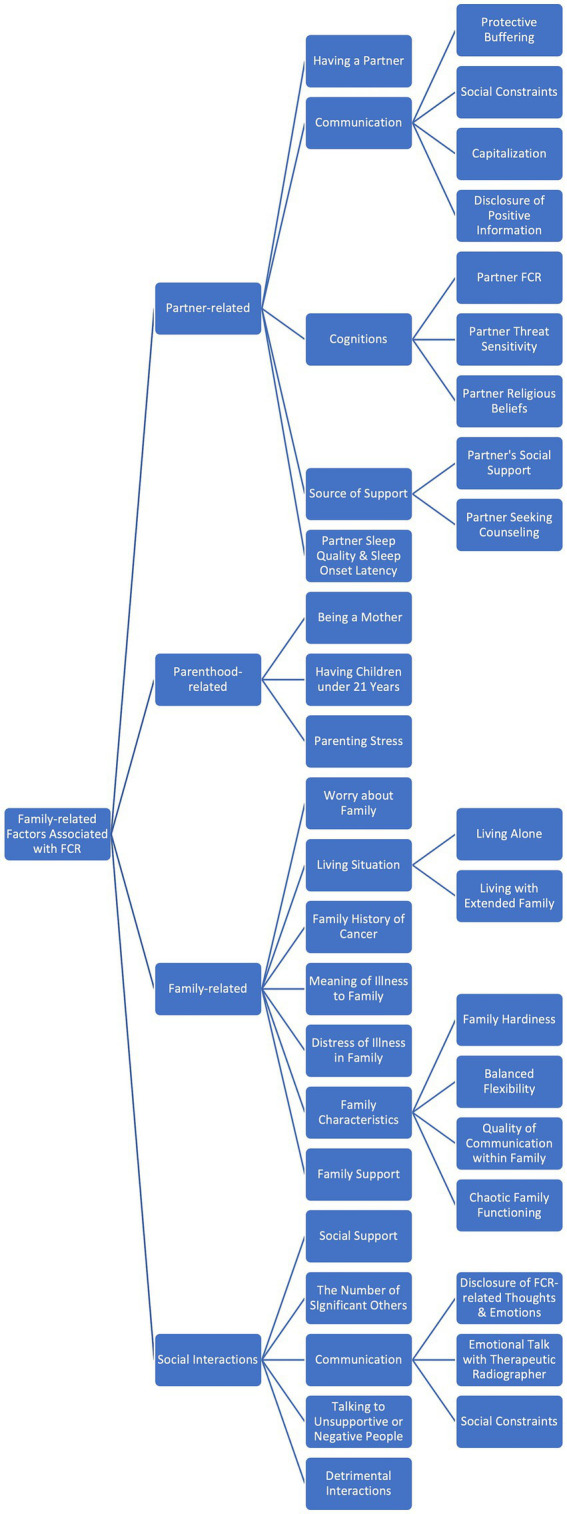
The Categorisation of family-related factors associated with FCR (Fear of Cancer Recurrence).

Researchers in the FCR field have addressed many family-related variables that have proved to be linked to FCR. However, most of them cannot be generalized to all CSs due to the dominance of research on women and cultural gulfs that are very important in family matters. Also, our knowledge of how parenthood, especially fatherhood, can affect the nature and intensity of FCR is extremely limited, which calls for further investigation. The current literature review attempted to critically analyse the most significant results of the previous studies on family-related factors associated with FCR and categorize them in a way that reveals the strengths and limitations of the current models and sets the stage to elaborate on them (please see Figure 1 for an overview).

## 5 Conclusion

In brief, we categorized family-related factors associated with survivor FCR into partner-related factors, including subgroups of disclosure to partner, cognitions of partner, and partner’s sources of support; parenthood-related factors, including having children and parenting stress; family-related factors, including living situation, family history of cancer, family’s perception of the illness, and family characteristics; and social interactions including social support, disclosure, social constraints, and attitudes of others. Knowing how and why each factor relates to survivor FCR helps us to construct a more comprehensive family-based model in completion of Mellon et al.’s (2007) model, which can, in turn, assist clinicians in designing family interventions for managing FCR. Researchers in the FCR field have addressed many family-related variables that have proved to be linked to FCR. However, most of them cannot be generalized to all CSs due to the dominance of research on women and cultural gulfs that are very important in family matters. Also, our knowledge of how parenthood, especially fatherhood, can affect the nature and intensity of FCR is extremely limited, which calls for further investigation. The current literature review attempted to critically analyse the most significant results of the studies on family-related factors associated with FCR and categorize them in a way that reveals the strengths and limitations of the current literature.

## Author contributions

AF: Conceptualization, Investigation, Writing – original draft, Writing – review & editing. MD: Conceptualization, Investigation, Methodology, Writing – original draft, Writing – review & editing. AK: Writing – original draft, Writing – review & editing.
